# COVID-19-Related Predictors of Fear and Attitude to Vaccination Displayed by Polish Students

**DOI:** 10.3390/vaccines10091524

**Published:** 2022-09-14

**Authors:** Anna Bartosiewicz, Edyta Łuszczki, Adam Bartosiewicz, Katarzyna Dereń, Łukasz Oleksy, Artur Stolarczyk

**Affiliations:** 1 Institute of Health Sciences, College of Medical Sciences, University of Rzeszow, 35-959 Rzeszow, Poland; 2 Faculty of Medicine, Medical Department, Medical University of Warsaw, 02-097 Warsaw, Poland; 3 Oleksy Medical & Sports Sciences, 37-100 Łańcut, Poland; 4 Orthopedic and Rehabilitation Department, Medical Faculty, Medical University of Warsaw, 02-091 Warsaw, Poland

**Keywords:** COVID-19, vaccine, fear, attitude, predictors, polish students

## Abstract

Vaccines are one of the most important achievements of modern medicine in maintaining the health of the population. The prolonged pandemic and subsequent lockdowns meant that the new COVID-19 vaccine was regarded by scientists and society as the way to end the pandemic and return to normal life. The purpose of this study was to analyze the factors responsible for the feeling of fear due to COVID-19 infection and the attitudes of medical students towards vaccination against COVID-19. A cross-sectional study was conducted online among medical students using standardized questionnaires: the Fear of COVID-19 scale and the Vaccination Attitude Examination scale. According to the results obtained, the respondents had a low level of fear of COVID-19 and the majority had positive attitudes towards vaccination against COVID-19. Regression analysis showed that the main predictors of fear of the pandemic and attitudes towards vaccination were age, sex, field of study, and sources of knowledge about vaccines. The analysis of factors related to the discussed issues can be the basis to formulate educational and preventive programs, to shape positive attitudes of future health sector employees toward the issue of preventive vaccination, as well as for the development of strategies to promote vaccination against COVID-19.

## 1. Introduction

The world was horrified by the news about the new COVID-19, which not only deprived millions of people of their health and lives, but also affected our daily functioning [1,2,3,4]. The prolonged pandemic and subsequent lockdowns meant that the new COVID-19 vaccine was regarded by scientists and society as the way to end the pandemic and the return to normal life [5,6]. Vaccines are one of the most important achievements of modern medicine for maintaining the health of the population [7,8]. They help to control the spread of infectious diseases and there is ample evidence that they are safe and effective [9,10,11]. However, for this method to be effective, public acceptance of vaccines and willingness of people to get vaccinated are necessary [8,12]. For many years, the WHO has indicated the need for vaccination as a factor to prevent the occurrence of many dangerous infectious diseases [13]. At the beginning of 2021, the emergence of COVID-19 vaccines produced by various pharmaceutical companies caused a great deal of controversy and a cultural, rather than scientific, debate, which largely blocked rational and scientific arguments [5,14]. The WHO has approved several vaccines manufactured by various companies [15,16,17,18]. The new vaccines had both many supporters and opponents [19]. The first professional group that had access to the new vaccine was, inter alia, medical professionals, including students from medical faculties [20,21]. Compared to other European countries, Poland has a low number of people willing to receive the COVID-19 vaccine [22,23]. This antivaccine attitude is considered by the WHO to be the main threat to global health and the spread of infectious diseases, despite the availability of effective vaccines [24]. On the other hand, a positive attitude towards the whole issue of preventive vaccination can be a predictor of a positive attitude toward vaccination against COVID-19 [25,26]. An individual’s attitude towards specific situations may be shaped by emotional, cognitive, and behavioral factors [27]. Those factors should be taken into account when analyzing factors that influence attitudes to vaccination against COVID-19 [28]. Research on attitudes towards preventive vaccinations conducted before the COVID-19 pandemic showed that a positive attitude in this regard depends on the age of the respondents (older people are more likely to receive vaccinations), knowledge in this field, and reliable information provided by medical personnel, especially physicians [27,29]. On the other hand, research carried out during the COVID-19 pandemic has shown that it is important to educate the public, to be aware of the benefits of vaccination, or that certain persons belonging to specific risk groups are at higher risk of contracting COVID-19 [27]. Other researchers indicate that people who were afraid that their relatives would get COVID-19 were more likely to receive the vaccine [30,31]. Furthermore, the belief in vaccine conspiracy theories and the confidence that COVID-19 is just a figment of pharmaceutical companies have shaped negative attitudes towards immunization [32,33].

The Max Planck Institute conducted a Survey on Health, Aging, and Retirement in Europe among more than 47,000 respondents from 27 European countries and Israel. It has been shown that the healthier a person is, the more likely they are to be negative about vaccination against COVID-19. As many as half of the respondents declared that they would rather not get vaccinated and 18% were completely sure that they would never get vaccinated. This attitude was mostly (54%) represented by the inhabitants of Romania. The above report also highlights factors such as sex and age. Younger people are more likely to have negative attitudes towards vaccination. In terms of sex, women are more likely to be undecided or refuse vaccines than men, with the exception of Hungary, Switzerland, and Portugal, where, in turn, more men were not vaccinated. According to data from the Max Planck Institute, experiencing the disease caused by COVID-19 yourself or among family and friends, especially with a severe course, may be a factor in determining attitudes towards immunization [34]. Research shows that another factor can be people’s trust in health professionals who, during their work with COVID-19 patients, can educate them and thus shape their attitudes towards vaccination [35,36,37,38,39].

Medical students, as future health professionals, can set an example for the rest of society in this area. Understanding the attitudes of medical students in the field of immunization may be the basis for education and raising the awareness of future leaders and educators in the conduct of preventive health-related activities [40]. The purpose of the study was to analyze the factors responsible for the feeling of fear due to COVID-19 infection and the attitudes of medical students towards the COVID-19 vaccination.

## 2. Materials and Methods

### 2.1. Participants

Six hundred and fourteen medical students voluntarily completed the online questionnaire. Participants were recruited by sending a link using social media, such as Facebook or Twitter, to participate in the survey and using the snowball method. Participation in the study was voluntary, anonymous, and free of charge. Before completing the questionnaire, participants received a detailed description of the purpose of the study and instructions on how to complete the questionnaire. The survey was conducted in the period from January to April 2022, i.e., at the time when most universities were returning to full-time education. Medical students were required to take the COVID-19 vaccination or undergo permanent tests to be able to participate in practical classes at medical institutions. Data were collected using an electronic form (Google Forms).

### 2.2. Questionnaire

The questionnaire included questions about the sociodemographic data of students (age, sex, field of study, sources of knowledge about COVID-19, and questions prepared on the basis of a literature review on vaccination issues and related to the COVID-19 pandemic: Question: Have you been infected with COVID-19 in the last year? Answer: No or Yes, if yes, please specify the course of the disease: mild, moderate, severe. Question: Have your family/close friends been infected with COVID-19 during the last year? Answer: No or Yes, if yes, please specify the course of the disease: mild, moderate, severe. Question: Do you follow the current vaccination schedule? Do you think vaccinations, and especially vaccinations against COVID-19, should be compulsory? Do you think that the COVID-19 vaccines available on the market are safe and effective? Should you get vaccinated with them? Answer: according to the five-point Likert scale: definitely yes, yes, I have no opinion, no, definitely not. Additionally, two standardized scales were used: Vaccination Attitudes Examination Scale (VAX) and Fear of COVID-19 Scale (FCV-19S).

#### 2.2.1. Vaccination Attitudes Examination Scale (VAX)

The VAX scale consists of 12 items and examines attitudes towards immunization. It allows for the assessment of the general attitude towards vaccination, along with four subscales. Each subscale of the VAX scale consists of 3 questions scored from 1 to 4 points, and hence, each scale has a range of values from 3 to 18 points. The total score is a number between 12–72 points. The higher the number of points, the greater the intensity of the attitude. For each of the subscales and the VAX total score, the average number of points per question is calculated, which is interpreted according to the single question key where: 1 means that the given attitude is definitely not present; 2 means that the given attitude does not exist; 3 means that the given attitude is rather absent; 4 means that the attitude is rather present; 5 means that the given attitude is present; 6 means that the attitude is definitely present. After reverse coding, the scale and subscale results are produced by averaging the respective elements: Subscale 1: lack of confidence in the benefits of the vaccine (questions 1, 2 and 3); Subscale 2: concerns about unforeseen future effects (questions 4, 5 and 6); Subscale 3: concerns about trade speculation (questions 7, 8 and 9); Subscale 4: preference for natural immunity (questions 10, 11 and 12) [41].

#### 2.2.2. Fear of COVID-19 Scale (FCV-19S)

The FCV-19S scale consists of seven statements that describe the fears of the respondent related to COVID-19 infection and allows for assessment of the level of anxiety due to COVID-19 infection. The scale score is a number between 7–35 points, and higher numbers indicate a higher level of anxiety. The average number of points per question is calculated and interpreted according to the key for a single question, according to which: 1 means no fear, 2 means no fear, 3 is a neutral answer, 4 is fear, and 5 is definitely fear [42]. The VAX and FCV-19S scales have been adapted to Polish conditions and their use was approved by the authors of the scale [43].

### 2.3. Ethics

The study was approved by the Institutional Bioethics Committee of Rzeszow University (Resolution No. 05/05/2021) and all relevant administrative bodies.

### 2.4. Statistical Analysis

The analysis of quantitative variables (i.e., expressed in number) was performed by calculating the arithmetic mean, standard deviation, median, and quartiles. The analysis of qualitative (i.e., non-numeric) variables was performed by calculating the number and percentage of occurrences of each value. One- and multivariate analysis of the influence of many variables on the quantitative variable was performed using the linear regression method. The results are presented as values of the regression model parameters with a 95% confidence interval.

A significance level of 0.05 was adopted in the analysis. Thus, all p values below 0.05 were interpreted as showing significant relationships. The analysis was performed using the R software, version 4.1.3 [44].

## 3. Results

### 3.1. Characteristics of the Study Group

Six hundred and fourteen students participated in the study, the average age of the respondents was 21 years, and the vast majority of the respondents were women (83.39%). The respondents are students of medical faculties, mainly dietetics (37.13%) and medicine (34.2%). The main source of knowledge for the respondents was the Internet (69.06%), followed by the scientific literature (51.47%), and a doctor (50.81%). Five hundred and fourteen students (83.71%) declared compliance with the current vaccination schedule (definitely yes and yes). The belief that vaccination, particularly against COVID-19, should be mandatory (definitely yes and yes) was declared by 244 (39.74%) people. The belief that the COVID-19 vaccines available on the market are safe and should be used as a means of immunization was declared (definitely yes and yes) by 335 (54.56%) students. Two hundred and nineteen students reported having had a COVID-19 infection in the last 12 months, of which 148 were mild, 48 were moderately severe, and 23 were severe. COVID-19 infection in a family member during the last 12 months was declared by 436 people, of which 173 had a mild course, 181 had a moderate course of the disease, and 82 had a severe course (Table 1).

### 3.2. Fear of COVID-19 Scale

The mean FCV-19S score was 14.04 points, or about 2.01 points per round question. According to the results obtained, the respondents were not afraid of COVID-19 (Table 2).

One-way analysis of the linear regression model (a model separate for each of the analyzed features) showed that the significant predictors (*p* < 0.05) of the FCV19-S score are age (0.309), male gender (−2.888), field of study, sources of knowledge about COVID-19, attitude towards the mandatory schedule of preventive vaccinations, the belief that vaccination against COVID-19 should be mandatory, the belief in the safety of vaccines, and having had COVID-19 disease, with a moderately severe or severe course.

The multivariate linear regression model showed that the significant independent predictors (*p* < 0.05) of the FCV19-S score were age, male gender, and the belief that vaccination, especially against COVID-19, should be mandatory (Table 3).

### 3.3. Vaccination Attitude Examination Scale (VAX)

The average score on the scale with respect to the attitude towards preventive vaccinations is 40.02 points, which gives 3.34 points per question (rounded 3), indicating a lack of a negative attitude of the respondents towards preventive vaccinations. The results of individual subscales also indicate a lack of a negative attitude of the students surveyed towards the immunization program (Table 4).

One-way linear regression analysis (a model separates for each of the analyzed features) showed that age, male sex, field of study, sources of knowledge about COVID-19, and adherence to the mandatory vaccination schedule are significant predictors (*p* < 0.05) of the scale result relating to belief in the effectiveness of the available vaccine against COVID-19, and attitude towards mandatory vaccinations, especially against COVID-19. The multivariate linear regression model showed that significant independent predictors (*p* < 0.05) of this scale score are age, gender, field of study, sources of knowledge about the COVID-19, attitudes towards compulsory vaccination, in particular against COVID-19, and belief in the safety of available on the market COVID-19 vaccines (Table 5).

## 4. Discussion

The purpose of the study was to analyze the factors responsible for the feeling of fear due to COVID-19 infection and the attitudes of medical students towards vaccination against COVID-19. The results obtained in terms of the participants’ experiences related to the COVID-19 pandemic showed that the main sources of knowledge about the pandemic were the Internet (69.06%), the scientific literature (51.47%), and a doctor (50.81%) in that order. Currently, the Internet has become a very popular place for searching for knowledge, including health knowledge. According to the BioStat^®^ Research and Development Center, more than half of Poles look for health information on the Internet; this especially applies to young women with higher education [45]. The results of a survey among medical students in Jordan showed that medical students used mainly social media (83.4%) and online search engines (84.8%) as their preferred source of information on COVID-19 and relied less on medical search engines (64.1%) [46]. Jarynowski et al. present similar results, indicating that the social interest of Internet users in topics related to the SARS-CoV-2 and COVID-19 during the pandemic in Poland is large and constitutes an important source of knowledge in this field [47]. Researchers indicate that the dissemination of inaccurate and false information on social media about COVID-19 may be one of the most important factors in negative attitudes towards vaccination against COVID-19. This really matters, especially among young adults, for whom the Internet is the main source not only of daily news, but scientific information and specialized knowledge on health as well [48,49,50,51].

Our study showed that the vast majority of students (83.71%; *N* = 514) declared (definitely yes and yes) adherence to the obligatory vaccination schedule. However, only 39.74% of the respondents declared the belief that vaccination, and in particular against COVID-19, should be mandatory (definitely yes and yes). Such an attitude may result from the specificity of studies and knowledge in this field. The correlations regarding the relationship between the level of education and the attitude presented towards immunization differ [27]. There are studies that show a negative relationship [52] and a positive relationship [53], as well as a complete lack of correlation in this respect [54]. Other studies indicate that the phenomenon of questioning both the need for and the safety of preventive vaccines is increasingly observed. The main reasons are low health awareness, intentional behavior resulting from individual lifestyle, religious beliefs, financial situation, or personal approach to health protection [55,56,57].

According to Ołpiński, a negative attitude towards vaccinations may be due to the fact that the current generation, especially the young, do not remember people dying or even suffering from infectious diseases [58]. Muller also claims that the decrease in confidence in vaccination is due to the lack of people’s experience of being ill and facing the consequences of having an infectious disease, while focusing more on the adverse events following immunization (AEFI) than on the benefits of vaccination [59]. Ołpiński also points out that the sources of concern for the US population regarding vaccine safety are due to the activity of antivaccine movements, distrust of the government and health care institutions, the influence of the mass media (mainly the Internet), propagation of conspiracy theories about the financial ties between the government, pharmaceutical companies and doctors, and lack of complete scientific knowledge in the field of etiology of many diseases [56,58]. Furthermore, young people are concerned about the immediate and long-term side effects of vaccines, especially those related to fertility [60,61]. The positive attitude towards vaccinations obtained in our study may result from the fact that these are students of medical faculties, whose universities often make continuing their education dependent on being vaccinated. Although in Poland there are currently no legal tools allowing universities to enforce vaccinations, the government is considering legal regulations in this area [62]. Additionally, more than half of our respondents (54.56%) declared that they considered the current COVID-19 vaccines safe. According to the results of Papaginnis et al. and Szmyd et al., fear of side effects from vaccination is a negative correlate of readiness for vaccination [30,31], despite the fact that the WHO provides specific confirmations on the safety of COVID-19 vaccines [63]. On the basis of numerous studies, it can be said that the available vaccines are safe for young people and even pregnant women [64,65,66,67,68,69,70,71,72]. However, only upcoming long-term research in this area will be more credible and convincing [66].

A factor that may influence attitudes towards vaccination is personal experience of the disease, its course, or illness among family members or friends. It can also be a concern for relatives, especially the elderly and those at risk [20]. As Manning points out, this attitude was presented by almost 70% of students participating in the survey in this area [20].

In our study, 219 students declared COVID-19 infection in the last 12 months, of which 148 were mild, 48 were moderately severe, and 23 were severe. COVID-19 infection in a family member during the last 12 months was declared by 436 people, of which 173 had a mild course, 181 had a moderate course of the disease, and 82 had a severe course.

According to Gotlib et al., personal and indirect experiences with COVID-19 and the desire to prevent relatives from developing the disease may be an important factor in positive attitudes toward vaccination [73]. On the other hand, a study by Jach et al., conducted before massive COVID-19 vaccination in Poland found no association between attitudes toward the COVID-19 vaccine after contracting the disease in person or through relatives and having loved ones who died from COVID-19. The need to remain in quarantine by study participants or their families was also not a predictor. Therefore, the results obtained led the researchers to reject the hypothesis that the personal experience of the consequences of the COVID-19 pandemic is associated with presentation of a positive attitude towards vaccination [27]. Jach et al., argue that when looking for factors relevant to attitudes toward COVID-19 vaccines, the focus should be on other aspects, such as psychological or socio-environmental factors [27].

The results of the FCV-19S scale showed that the respondents of our study were not concerned about the COVID-19 (14.04 out of the 35 possible points). This may be due to the fact that the study group consisted of young people whose immunity, even in the face of infection, will allow for a safe course of the disease. However, since the beginning of the pandemic, the WHO has pointed out that both the mere fact of a pandemic and the restrictions that result from it can cause stress, and therefore, cause a feeling of anxiety [74]. Doshi et al., obtained similar results. Most of the respondents in the Indian population (54.8%) showed a low level of anxiety due to COVID-19 [75]. In contrast, in the study by Elsami et al., the level of fear of COVID-19 among Indian radiologists, also assessed with the FCV-10S scale, was significantly higher and was 24.7 out of 35 possible points [76]. A systematic review by Quadros et al., showed that the majority of respondents to the studies analyzed were concerned about being infected with the COVID-19. This mainly concerned women, young people, city dwellers, divorced couples, health care workers, people in quarantine, and individuals with mental health problems [77]. A study conducted on a representative group of U.S. citizens (*N* = 10,368) showed that there is a high concern among respondents about COVID-19 and it amounts to approximately 7 out of 10, which is related to the incidence of the disease in a given case [78]. In a study by Al-Rahini et al., in the Saudi Arabian population, the mean FCV-19S score was 17.40 ± 5.751, with scores ranging from 7 to 35, indicating that participants in this study experienced significant levels of anxiety about COVID-19. More than 40% of the participants declared that they felt discomfort just thinking about COVID-19. More than a third (35.6%) of the participants said that they felt upset or anxious when they saw news stories and articles about COVID-19 on social networks. Additionally, 28.3% agreed that they are most afraid of COVID-19. The results obtained may be due to the fact that the study group was very diverse in terms of age (from 0 to 80 years) and the average age of the respondents was much higher (36.4–39.0) [79].

One-way analysis of the linear regression model showed that the significant predictors of the FCV19-S score are age, male sex, medical studies, sources of knowledge about COVID-19, attitude to the mandatory schedule of preventive immunization, the belief that vaccination against COVID-19 should be mandatory, belief in vaccine safety, and COVID-19 with moderate to severe course. On the contrary, the multivariate linear regression model showed that age, gender, and the belief that vaccination, especially against COVID-19, should be mandatory are important independent predictors of the FCV19-S score. The results of the survey conducted by Długosz showed that fear of COVID-19 is correlated with age, financial status, fear of loss of employment, mindset change index, and life position decrease index. Stress, the level of interest in the media, and the fear of becoming infected have a stronger impact [80]. Another study among college students identified demographic factors (age and gender) as the major predictors of fear of COVID-19. On the contrary, other studies conducted in the student population, in this regard, have shown that the fear of COVID-19 is stronger among women and increases with the age of the respondents [81,82,83].

Our results showed that the surveyed students showed a positive attitude towards immunization against COVID-19. Other researchers obtained similar results in this regard, and students, especially from medical faculties, generally have a positive attitude towards vaccination. Such results may be due to the high level of knowledge of these people and high health awareness [84,85,86]. However, the results concerning the relationship between a high level of knowledge and a positive attitude towards vaccination are heterogeneous [27] and indicate no positive correlation in this respect [54].

In addition, in some medical facilities, only fully vaccinated students are allowed to pursue practical activities in a hospital, which will allow them to complete their studies. Although students are not forced, but only encouraged to take the vaccine, according to Gotlib et al., the willingness to complete studies may be an important predictor of the attitude of medical students towards vaccination against COVID-19 [73]. Moreover, other researchers indicate that medical students have a more positive attitude towards vaccination against COVID-19 than towards other vaccinations [86]. In one of the largest studies conducted on this topic in Slovakia among more than 12,000 students, more than half (59%) had a positive attitude towards immunization [87].

Furthermore, univariate regression analysis showed that age, male sex, field of study, sources of knowledge of the COVID-19, adherence to the mandatory vaccination schedule, belief in the effectiveness of the available COVID-19 vaccine, and attitudes toward mandatory vaccination, in particular against COVID-19, are significant predictors of this scale score, while the multivariate linear regression model indicates that age, gender, field of study, sources of knowledge about the COVID-19, attitudes about compulsory vaccinations, in particular against COVID-19, and the belief about the safety of vaccines available on the market are important predictors of attitudes of medical students to COVID-19 vaccines. Other studies have shown that sex is an important predictor of attitudes towards COVID-19 vaccines and, as in our study, this attitude was more represented by men [27,88,89]. Funk et al., and Wang et al., point to a strong correlation between generally positive attitudes toward other vaccinations, including influenza vaccination, and vaccination against COVID-19 [90,91].

Petravić et al., based on the obtained results, showed that the male sex and age of the respondents were an important predictor of a positive attitude toward vaccination against COVID-19 (the older the respondents, the more willing). Furthermore, students of medicine are more positive about the topic than nurses. Researchers also point to hospital stay or death of relatives and the level of trust in experts, institutions, and vaccines as important factors [87]. Another study conducted in this field among medical workers and medical students showed that the vast majority of the respondents (82%) had a positive attitude towards vaccination against COVID-19. This attitude was presented by students of medicine rather than nurses, the predictor of a positive attitude was age, the younger the respondents, the more positive the attitude towards vaccination against COVID-19 they had [86]. Other studies also indicate that the positive attitude of students towards the vaccination against COVID-19 also depend on the university and the good organization of the recruitment and vaccination process itself [30,73,92].

Our study, conducted using standardized questionnaires (FVC-19S and VAX scale), raises a very important issue. Due to the constantly high number of cases of COVID-19 and complications resulting from this disease, it is important to know the attitudes of future health workers toward both fear of the COVID-19 pandemic and attitudes toward vaccination against COVID-19. Identification of factors relating to the discussed issues can be the basis for the development of educational and preventive programs, and for shaping positive attitudes of future doctors, nurses, and other health sector workers towards the issue of preventive vaccinations. The study has some limitations. As a cross-sectional study design, we are limited to a specific time frame and are not able to estimate how the level of fairness of COVID-19 and attitudes towards vaccination change over time. The study group concerned students of medical faculties; in further research, students of other nonmedical faculties should also be included in the study group. Moreover, possible response bias as most of the respondents were women (more than 4 every 5 respondents).

## 5. Conclusions

Our study showed that the level of anxiety due to COVID-19 among students was low and that they have a mainly positive attitude towards COVID-19 vaccines. The predictors responsible for the above-described issue include age, sex, type of study, sources of knowledge about the COVID-19, compliance to the obligatory vaccination schedule, belief in the effectiveness and safety of the available vaccine, and attitude towards other compulsory vaccinations. Due to the fact that the Internet and social media are the main source of knowledge for young adults, it is of paramount importance to provide credible and reliable vaccine information on the Internet. It is important to keep such information frequently updated as well. Therefore, a proper response to disinformation about vaccinations on the Internet may reduce vaccine hesitancy and minimize the loss of life. At the present stage of the pandemic, there are already many studies on the results of research on the factors that influence attitudes and intentions to adopt the COVID-19 vaccination. These include sociodemographic, health-related, and highly emotional psychological factors, such as individual risk of infection, disease, and even hospitalization or death.

Most of the results of our study confirm the findings of other researchers in this regard. Identifying the factors responsible for feeling anxious about COVID-19 can be used to formulate support programs for future health workers. These results can be very useful for the staff responsible for educating students, those responsible for the organization and implementation of vaccination programs, and for developing strategies to promote vaccination against COVID-19.

## Figures and Tables

**Table 1:** Characteristics of the study group.

Variable		Total (*N* = 614)
Age [years]	Average ± SD	22.85 ± 4.88
	Median	21
	Quartiles	20–23
Sex	Female	512 (83.39%)
	Male	102 (16.61%)
Field of study	Dietetics	228 (37.13%)
	Physiotherapy	79 (12.87%)
	Medical	210 (34.20%)
	Nursing/Obstetrics	97 (15.80%)
Information sources about vaccination *	Internet	424 (69.06%)
	Physician	312 (50.81%)
	Scientific literature	316 (51.47%)
	Nurse/Midwife	83 (13.52%)
	Friends/Family	178 (28.99%)
	Others	15 (2.44%)
	I’m not looking for information on this	8 (1.30%)
Adherence to the obligatory vaccination schedule	Definitely not	9 (1.47%)
	Not	35 (5.70%)
	I have no opinion	56 (9.12%)
	Yes	227 (36.97%)
	Definitely yes	287 (46.74%)
Belief that vaccination, especially against COVID-19, should be mandatory	Definitely not	102 (16.61%)
	Not	149 (24.27%)
	I have no opinion	119 (19.38%)
	Yes	116 (18.89%)
	Definitely yes	128 (20.85%)
Belief that COVID-19 vaccines on the market are safe and should be obligatory	Definitely not	40 (6.51%)
	Not	73 (11.89%)
	I have no opinion	166 (27.04%)
	Yes	206 (33.55%)
	Definitely yes	129 (21.01%)
COVID-19 infection in the last 12 months	No	395 (64.33%)
	Yes, mild disease course	148 (24.10%)
	Yes, moderate disease course	48 (7.82%)
	Yes, severe disease course	23 (3.75%)
COVID-19 infection in a family member in the last 12 months	No	178 (28.99%)
	Yes, mild disease course	173 (28.18%)
	Yes, moderate disease course	181 (29.48%)
	Yes, severe disease course	82 (13.36%)

* The values do not add up to 100 as multiple choice was possible.

**Table 2:** Fear of COVID-19 Scale–scores (*N* = 614).

**Point Range**	***N***	**M**	**SD**	**Mean per Question**	**Me**	**Min**	**Max**	**Q1**	**Q3**
5–35	614	14.04	5.09	2.01	14	7	35	10	17
**Point range**									
5–35									

Q1—quartile I; Q3—quartile III; M—arithmetic mean; SD—standard deviation; Me—Median; Min—Minimum; Max—Maximum.

**Table 3:** Factors influencing the level of anxiety due to COVID-19 infection (Fear of COVID-19 Scale). Single and multivariate linear regression model.

Variables		One-Factor Models				Multivariate Model			
		Parameter	95%CI		*p*	Parameter	95%CI		*p*
Age	Years	0.309	0.23	0.388	<0.001 *	0.24	0.156	0.324	<0.001 *
Sex	Female	ref.				ref.			
	Male	−2.888	−3.946	−1.831	<0.001 *	−2.14	−3.237	−1.044	<0.001 *
Field of study	Dietetics	ref.				ref.			
	Physiotherapy	−1.906	−3.188	−0.625	0.004 *	−0.927	−2.184	0.331	0.149
	Medical	−0.478	−1.417	0.461	0.318	−0.806	−1.815	0.202	0.117
	Nurse/Midwife	1.566	0.375	2.756	0.01 *	0.488	−0.725	1.701	0.431
Information sources about vaccination: Internet	No	ref.				ref.			
	Yes	−1.038	−1.905	−0.171	0.019 *	0.112	−0.759	0.983	0.802
Information sources about vaccination: Physician	No	ref.				ref.			
	Yes	1.276	0.477	2.075	0.002 *	0.688	−0.144	1.519	0.106
Information sources about vaccination: Scientific literature	No	ref.				ref.			
	Yes	0.656	−0.148	1.46	0.11	0.149	−0.62	0.918	0.705
Information sources about vaccination: Nurse/Midwife	No	ref.				ref.			
	Yes	2.397	1.234	3.559	<0.001 *	0.677	−0.529	1.883	0.271
Information sources about vaccination: Friends/Family	No	ref.				ref.			
	Yes	−0.011	−0.898	0.877	0.981	0.281	−0.588	1.149	0.527
Information sources about vaccination: Others	No	ref.				ref.			
	Yes	−0.447	−3.055	2.161	0.737	−0.257	−2.732	2.219	0.839
Information sources about vaccinations: I’m not looking for information on this	No	ref.				ref.			
	Yes	−2.316	−5.862	1.23	0.201	−0.47	−3.977	3.036	0.793
Adherence to the obligatory vaccination schedule	Definitely not	ref.				ref.			
	Not	4.025	0.338	7.712	0.033 *	0.428	−3.358	4.214	0.825
	I have no opinion	4.45	0.908	7.993	0.014 *	0.735	−2.956	4.427	0.696
	Yes	4.913	1.56	8.266	0.004 *	−0.01	−3.553	3.533	0.996
	Definitely yes	5.766	2.427	9.106	0.001 *	0.247	−3.296	3.79	0.891
Belief that vaccination, especially against COVID-19, should be mandatory	Definitely not	ref.				ref.			
	Not	2.561	1.324	3.797	<0.001 *	1.685	0.381	2.989	0.012 *
	I have no opinion	2.853	1.555	4.151	<0.001 *	1.843	0.369	3.317	0.015 *
	Yes	4.296	2.99	5.602	<0.001 *	3.129	1.562	4.697	<0.001 *
	Definitely yes	3.767	2.49	5.043	<0.001 *	3.218	1.491	4.945	<0.001 *
Belief that COVID-19 vaccines on the market are safe and should be obligatory	Definitely not	ref.				ref.			
	Not	1.995	0.064	3.926	0.043 *	1.29	−0.676	3.257	0.199
	I have no opinion	3.116	1.387	4.845	<0.001 *	1.719	−0.172	3.61	0.075
	Yes	3.613	1.917	5.309	<0.001 *	1.672	−0.317	3.66	0.1
	Definitely yes	3.779	2.002	5.555	<0.001 *	1.784	−0.438	4.006	0.116
COVID-19 infection in the last 12 months	No	ref.				ref.			
	Yes, mild disease course	−0.217	−1.172	0.738	0.656	−0.611	−1.563	0.341	0.209
	Yes, moderate disease course	1.697	0.182	3.212	0.029 *	0.99	−0.476	2.456	0.186
	Yes, severe disease course	2.395	0.269	4.521	0.028 *	0.556	−1.496	2.609	0.595
COVID-19 infection in a family member in the last 12 months	No	ref.				ref.			
	Yes, mild disease course	0.637	−0.426	1.701	0.241	0.342	−0.712	1.396	0.525
	Yes, moderate disease course	0.849	−0.202	1.9	0.114	0.113	−0.925	1.15	0.832
	Yes, severe disease course	1.215	−0.115	2.544	0.074	0.196	−1.081	1.473	0.764

* Statistically significant relationship (*p* < 0.05).

**Table 4:** Attitudes towards vaccination (VAX scale)–scores *N* = 614.

VAX (*N* = 614)	The Range of Values	Mean	SD	Average per Question	Me	Min	Max	Q1	Q3
Negative Attitude Toward Vaccination (VAX Total Score)	12–72	40.02	12.87	3.34	40	12	72	31	47
Lack of faith in vaccine effectiveness	3–18	9.34	4.44	3.11	9	3	18	6	12
Fear of unforeseeable consequences in the future	3–18	11.88	3.74	3.96	12	3	18	10	15
The belief that the goal of vaccination is the profit of the vaccine manufacturer	3–18	10.25	3.95	3.42	10	3	18	8	13
Prefer natural immunity	3–18	8.55	3.77	2.85	9	3	18	6	11

Q1—quartile I; Q3—quartile III; M—arithmetic mean; SD—standard deviation; Me—Median; Min—Minimum; Max—Maximum.

**Table 5:** Factors influencing attitudes towards vaccination (VAX scale). Single and multivariate linear regression model.

Variables		One-Factor Models				Multivariate Model			
		Parameter	95%CI		*p*	Parameter	95%CI		*p*
**Age**	**Years**	0.214	0.006	0.422	0.045 *	0.212	0.066	0.358	0.005 *
Sex	Female	ref.				ref.			
	Male	−6.976	−9.656	−4.296	<0.001 *	−2.299	−4.208	−0.39	0.019 *
Field of study	Dietetics	ref.				ref.			
	Physiotherapy	−0.404	−3.55	2.743	0.802	0.005	−2.183	2.194	0.996
	Medical	−8.637	−10.942	−6.331	<0.001 *	−2.19	−3.945	−0.435	0.015 *
	Nursing Obstetrics	−2.373	−5.295	0.549	0.112	−1.903	−4.014	0.208	0.078
Information sources about vaccination: Internet	No	ref.				ref.			
	Yes	1.268	−0.933	3.469	0.259	0.192	−1.324	1.708	0.804
Information sources about vaccination: Physician	No	ref.				ref.			
	Yes	−5.064	−7.061	−3.067	<0.001 *	−0.463	−1.911	0.985	0.531
Information sources about vaccination: Scientific literature	No	ref.				ref.			
	Yes	−4.487	−6.494	−2.481	<0.001 *	−1.053	−2.392	0.286	0.124
Information sources about vaccination: Nurse/Midwife	No	ref.				ref.			
	Yes	−1.197	−4.174	1.781	0.431	0.609	−1.49	2.708	0.57
Information sources about vaccination: Friends/Family	No	ref.				ref.			
	Yes	3.758	1.533	5.982	0.001 *	2.019	0.507	3.531	0.009 *
Information sources about vaccination: Others	No	ref.				ref.			
	Yes	1.002	−5.595	7.598	0.766	3.824	−0.485	8.134	0.083
Information sources about vaccinations: I’m not looking for information on this	No	ref.				ref.			
	Yes	−9.269	−18.22	−0.317	0.043 *	−10.883	−16.986	−4.78	0.001 *
Adherence to the obligatory vaccination schedule	Definitely not	ref.				ref.			
	Not	−2.241	−10.846	6.364	0.61	4.692	−1.898	11.283	0.163
	I have no opinion	−5.984	−14.253	2.284	0.157	3.179	−3.246	9.605	0.333
	Yes	−13.401	−21.227	−5.576	0.001 *	1.339	−4.828	7.505	0.671
	Definitely yes	−19.05	−26.844	−11.256	<0.001 *	0.885	−5.282	7.053	0.779
Belief that vaccination, especially against COVID-19, should be mandatory	Definitely not	ref.				ref.			
	Not	−7.444	−9.871	−5.017	<0.001 *	−3.164	−5.434	−0.895	0.006 *
	I have no opinion	−13.321	−15.869	−10.772	<0.001 *	−5.74	−8.305	−3.174	<0.001 *
	Yes	−17.405	−19.968	−14.841	<0.001 *	−7.724	−10.452	−4.996	<0.001 *
	Definitely yes	−25.756	−28.263	−23.25	<0.001 *	−10.879	−13.885	−7.872	<0.001 *
Belief that COVID-19 vaccines on the market are safe and should be obligatory	Definitely not	ref.				ref.			
	Not	−5.648	−9.006	−2.29	0.001 *	−5.671	−9.094	−2.248	0.001 *
	I have no opinion	−15.401	−18.408	−12.395	<0.001 *	−13.343	−16.635	−10.051	<0.001 *
	Yes	−22.051	−25.001	−19.102	<0.001 *	−15.921	−19.383	−12.46	<0.001 *
	Definitely yes	−32.573	−35.662	−29.484	<0.001 *	−22.237	−26.105	−18.369	<0.001 *
COVID-19 infection in the last 12 months	No	ref.				ref.			
	Yes, mild disease course	0.159	−2.273	2.592	0.898	−0.266	−1.924	1.392	0.753
	Yes, moderate disease course	2.451	−1.407	6.309	0.214	1.233	−1.32	3.785	0.344
	Yes, severe disease course	−1.275	−6.689	4.139	0.644	2.159	−1.413	5.732	0.237
COVID-19 infection in a family member in the last 12 months	No	ref.				ref.			
	Yes, mild disease course	0.65	−2.045	3.344	0.637	−0.584	−2.419	1.251	0.533
	Yes, moderate disease course	−1.027	−3.691	1.638	0.45	−0.412	−2.218	1.395	0.655
	Yes, severe disease course	−0.995	−4.363	2.374	0.563	−1.137	−3.359	1.086	0.317

* Statistically significant relationship (*p* < 0.05).

## Data Availability

The data presented in this study are available by reasonable request from the corresponding author. The data are not publicly available due to restrictions, e.g., their containing information that could compromise the privacy of research participants.
